# Beyond the Usual Suspects: A Pictorial Case Report of Lemmel Syndrome Emphasizing the Role of Multimodality Imaging

**DOI:** 10.7759/cureus.60097

**Published:** 2024-05-11

**Authors:** Sakthi Ganesh Subramonian, Karpagam R K, Sam Raja, Ajay Lucas, Paarthipan Natarajan

**Affiliations:** 1 Department of Radio-Diagnosis, Saveetha Medical College and Hospital, Saveetha Institute of Medical and Technical Sciences (SIMATS) Saveetha University, Chennai, IND

**Keywords:** biliary stenting, endoscopic sphincterotomy, magnetic resonance cholangiopancreatography (mrcp), biliary obstruction, periampullary duodenal diverticulum, lemmel syndrome

## Abstract

Lemmel syndrome, characterized by biliary and pancreatic duct obstruction secondary to a periampullary duodenal diverticulum (PAD), remains a rare and often overlooked diagnosis. Although duodenal diverticula are found in approximately 23% of the population, only about 5% of these cases lead to complications such as obstructions. A new case of Lemmel syndrome is demonstrated in the article about a middle-aged woman with chronic epigastric pain and right upper abdominal quadrant initially misdiagnosed as cholelithiasis. The accuracy of diagnosis was made possible by multimodal imaging methods, such as ultrasound, magnetic resonance cholangiopancreatography (MRCP), and computed tomography (CT) with oral contrast when a diffuse common bile duct was compressed by a PAD. Additionally, it highlights the necessity of including Lemmel syndrome in cases where patients have dilated bile ducts without calculi or mass lesions while emphasizing advanced imaging techniques for the revelation of structural malformations that underlay these conditions. The endoscopic intervention was minimally invasive but effective in relieving symptoms through sphincterotomy followed by laparoscopic cholecystectomy and biliary stent placement, thus making a point of the need for multiple disciplinary approaches toward treatment rare phenomenon like this one.

This case report not only sheds light on the diagnostic and therapeutic avenues for Lemmel syndrome but also serves as a valuable educational resource for healthcare professionals. It emphasizes the need for heightened clinical vigilance and the adept use of imaging modalities in cases of biliary obstruction with obscure etiology. By contributing to the growing knowledge of this rare condition, we aim to facilitate timely diagnosis and optimize patient outcomes.

## Introduction

Lemmel syndrome, first described by Lemmel in 1934 [1], is a rare condition characterized by a periampullary duodenal diverticulum (PAD) causing extrinsic compression and obstruction of the biliary and pancreatic ducts [1-3]. It is important to note that while duodenal diverticula are relatively common, only about 5% of these cases result in the type of obstruction seen in Lemmel syndrome, underscoring its clinical rarity and the need for awareness among healthcare providers [2]. Its infrequent occurrence, diverse clinical presentations, and nonspecific symptoms often lead to considerable diagnostic delay, with many cases initially misattributed to more common biliary or pancreatic pathologies [1,4,5]. The limited literature on Lemmel syndrome, primarily consisting of case reports and small case series, highlights the need for comprehensive documentation and analysis of individual cases to enhance our understanding of its clinical spectrum, diagnostic challenges, and optimal management strategies [6,7].

In this report, we present the case of a middle-aged woman with chronic epigastric and right upper quadrant (RUQ) pain, initially diagnosed as cholelithiasis. An initial ultrasound showed a large gallstone within the gallbladder with no evidence of cholecystitis and a dilated common bile duct (CBD), suggesting a possible obstructive process more distally. This finding prompted further examinations using magnetic resonance cholangiopancreatography (MRCP) and computed tomography (CT) with oral contrast, which revealed a PAD compressing the distal CBD, thus establishing the diagnosis of Lemmel syndrome. The patient underwent successful endoscopic sphincterotomy, biliary stenting, and laparoscopic cholecystectomy to address both conditions.

This pictorial case report showcases the complementary strengths of various imaging modalities in diagnosing Lemmel syndrome and serves as a valuable educational resource for radiologists and other healthcare professionals. It emphasizes the importance of considering Lemmel syndrome in the differential diagnosis of biliary obstruction, particularly when initial investigations fail to identify an apparent cause [8,9]. Furthermore, this report underscores the critical role of a multidisciplinary approach involving radiologists, gastroenterologists, and surgeons in accurately diagnosing and effectively managing this rare condition [10,11]. By sharing our experience and insights, we aim to raise awareness of Lemmel syndrome, emphasize the need for heightened clinical suspicion, and promote a collaborative approach to ensure timely diagnosis and optimal patient outcomes.

## Case presentation

A woman in her 50s presented with complaints of epigastric and RUQ pain for the past year. Six months ago, she underwent an ultrasound scan that revealed cholelithiasis without cholecystitis. However, she did not seek further medical attention at that time. Her pain worsened significantly over the past two weeks, prompting her visit to our institution. Upon physical examination, the abdomen was found to be non-tender, and the absence of Murphy’s sign suggested a lack of gallbladder inflammation. Basic blood tests were normal except for a mildly elevated white blood cell count (12,700 cells/mm^3^) with an increased neutrophil count (9,300 cells/mm^3^). Her lipid profile was abnormal. She reported no significant medical history (comorbidities) other than a previous left cataract surgery.

**Figure 1:**
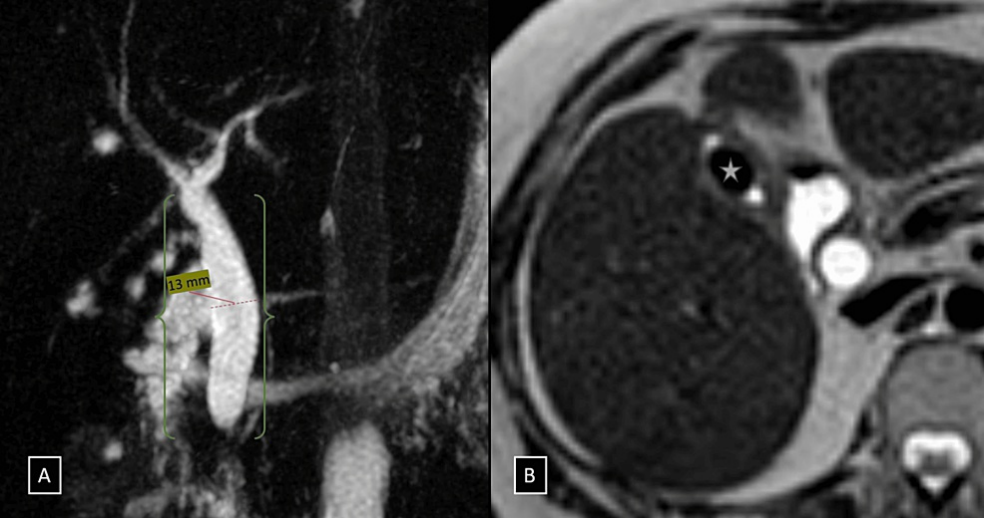
(A and B) Coronal MIP images from heavily T2-weighted pulse sequences showing a dilated CBD with abrupt tapering just proximal to the ampulla of Vater (denoted by a green brace in figure A). Axial sections of T2-weighted imaging showing a large ovoid calculus measuring 13 × 9 mm within the lumen of the gallbladder (white star in B). CBD, common bile duct

An initial ultrasound performed at our institution showed a gallstone with a “wall-echo-shadow sign” (indicating a possible stone within the gallbladder) and no surrounding inflammation. The CBD appeared dilated (13 mm), raising suspicion of obstruction. However, the ultrasound did not reveal any stones in the CBD or gross lesions in the pancreatic head. Therefore, an MRCP was recommended for further evaluation. The MRCP revealed a contracted gallbladder with a large ovoid flow void (~13 × 9 mm) within its lumen. The CBD was significantly dilated, measuring approximately 13 mm at its widest point, and exhibited abrupt tapering just proximal to the ampulla of Vater. Notably, there were no obstructing calculi or gross mass lesions identified in the distal CBD or pancreatic head, and the pancreatic duct appeared normal (Figure 1).

**Figure 2:**
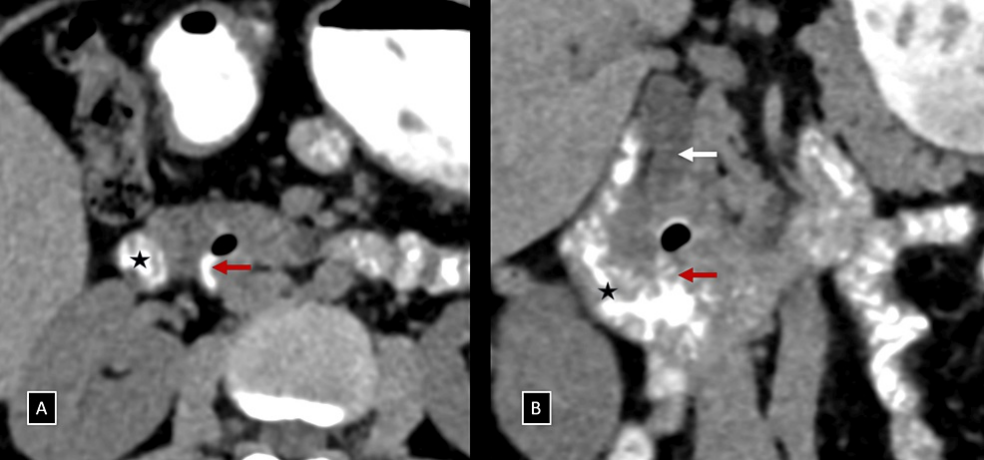
(A and B) Axial and coronal reformatted images of NCCT with positive oral contrast administration demonstrating a small periampullary diverticulum (red arrows) with internal air foci arising from the medial wall of the distal second part/proximal third part of the duodenum (black star), compressing the retro-pancreatic portion of the distal CBD extrinsically (white arrow), just proximal to the ampulla of Vater. NCCT, non-contrast enhanced computed tomography; CBD, common bile duct

**Figure 3:**
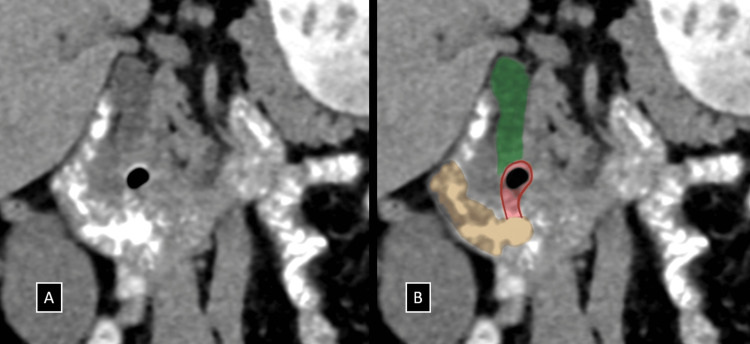
(A and B) Coronal reformatted images of NCCT with positive oral contrast administration (A) with accompanying annotated illustration (B) demonstrating the periampullary diverticulum (red highlight) with internal air foci arising from the medial wall of the distal second part/proximal third part of the duodenum (brown), compressing the retro-pancreatic portion of the distal CBD extrinsically (green), just proximal to the ampulla of Vater. NCCT, non-contrast enhanced computed tomography; CBD, common bile duct

To further investigate the cause of CBD dilatation in the absence of a distal calculus or a peri-ampullary mass, a low-dose CT scan was performed. The plain CT scan identified a suspicious periampullary region diverticulum containing air foci. Given this finding, Lemmel syndrome was suspected, and oral contrast was administered. Subsequent CT with oral contrast clearly demonstrated a small periampullary diverticulum (~11 mm) with internal air foci arising from the medial wall of the distal second part/proximal third part of the duodenum. This diverticulum was compressing the retropancreatic portion of the distal CBD extrinsically, just proximal to the ampulla of Vater (Figures 2, 3 and Video 1). Based on these findings, Lemmel syndrome was considered the primary differential diagnosis.

**Video 1:** Annotated video walkthrough demonstrating the periampullary diverticulum compressing the retro-pancreatic portion of the distal CBD extrinsically on axial reformatted images of NCCT with positive oral contrast administration. NCCT, non-contrast enhanced computed tomography; CBD, common bile duct

Endoscopic intervention confirmed the presence of a PAD visualized using a side-viewing endoscope. Upon the diagnosis of Lemmel syndrome complicated by cholelithiasis, the patient underwent a tailored treatment approach to address both conditions effectively. Initially, endoscopic retrograde cholangiopancreatography (ERCP) with sphincterotomy was performed to relieve the extrinsic compression on the distal CBD caused by the PAD. A stent was placed within the bile duct to ensure its patency and facilitate bile drainage. To address the gallstone, a laparoscopic cholecystectomy was simultaneously scheduled, aiming to remove the gallbladder and prevent future complications related to gallstones. Post-ERCP, the patient’s diet was adjusted to low fat to minimize biliary secretion and alleviate symptoms. The multidisciplinary approach led to significant symptomatic relief. The bile duct stent was removed after six weeks, and regular endoscopic surveillance was planned to monitor the periampullary diverticulum and ensure there were no further complications. This comprehensive approach addressed both the extrinsic compression from Lemmel syndrome and the cholelithiasis, providing immediate symptom relief but also aimed at preventing long-term recurrences.

## Discussion

Lemmel syndrome, characterized by a PAD causing obstruction and dilation of the biliary and pancreatic ducts, presents an interesting challenge in diagnosis and management due to its rarity and diverse manifestations. This condition, initially described by Lemmel in 1934 [1], often eludes standard diagnostic approaches and lacks a universally accepted treatment protocol, thereby making each case unique and requiring a tailored approach. This review synthesizes information from a case report alongside a comprehensive literature review, aiming to shed light on the diagnostic puzzles and therapeutic options for managing this syndrome.

The incidence of diverticula within the gastrointestinal tract is common, predominantly asymptomatic, and often discovered incidentally. While the colon is the most frequent location for diverticula, the duodenum, particularly its second portion, is the second most common site [5]. The unique subset of periampullary duodenal diverticula poses a significant risk by potentially compressing nearby structures, specifically the biliary and pancreatic ducts, leading to symptomatic presentations in a minority of patients. The hallmark of Lemmel syndrome is obstructive jaundice without the presence of a gallstone resulting from such compression [1-4].

There are several theories about the underlying causes of PAD and Lemmel syndrome. One suggestion is that chronic diverticulitis can lead to fibrosis, which in turn causes persistent inflammation and obstruction of the CBD [6]. Another possible cause is dysfunction of the sphincter of Oddi, which might result in mechanical blockage [7,8]. This diverticulum, when inflamed or enlarged, can compress the CBD, obstructing the flow of bile and causing jaundice and other obstructive symptoms.

While some individuals with Lemmel syndrome may not experience any symptoms, others may present with upper right quadrant abdominal pain, jaundice due to bilirubin buildup, nausea, vomiting, dark urine, pale stools, and occasionally fever. In a recent exhaustive literature review conducted by Love et al. [9], analyzing 17 cases, including the presented case, abdominal pain was the most common symptom, reported in 64.7% of cases, while fever was noted in 52.9%. Physical examination findings were documented in 94.1% of cases, with 43.8% showing abdominal tenderness predominantly localized to the RUQ. Jaundice was observed in 68.8% of cases, and laboratory data indicated an elevation in serum total bilirubin in 81.3% of cases, highlighting obstructive jaundice as a significant presentation of Lemmel Syndrome. Liver enzyme elevations were the second most common lab abnormality. These findings underscore the clinical variability in the presentation of Lemmel Syndrome, with a significant proportion of patients exhibiting symptoms of obstructive jaundice and abdominal pain, but also including cases with fever and other nonspecific symptoms. In the unusual case presented by the authors, the patient presented with gastric outlet obstruction and showed no visible signs of jaundice despite radiological evidence of biliary and pancreatic duct dilation. Since the symptoms can be non-specific, appropriate imaging techniques play a pivotal role in avoiding misdiagnosis of this condition.

Diagnosing Lemmel’s syndrome presents significant challenges. Various diagnostic tools can be employed, such as ultrasound, CT scans, MRI, and upper gastrointestinal endoscopy. However, the use of a side-viewing endoscope during ERCP is considered the most reliable method for identifying PAD and determining the cause of the obstruction, and it also provides a therapeutic procedure at the same time [8-10]. Diagnostic imaging typically reveals cavitary lesions with thin walls situated on the inner wall of the duodenum’s second segment, accompanied by dilation of both extrahepatic and intrahepatic biliary ducts [11]. These lesions, which may contain either gas or liquid, demand careful examination by radiologists to distinguish them from other conditions, such as pancreatic pseudocysts, abscesses, or cystic tumors in the head of the pancreas [12,13].

The treatment approach for Lemmel syndrome depends on the severity of the obstruction and the patient’s overall health. In many cases, endoscopic intervention, as performed in this case, is the preferred approach. This minimally invasive procedure involves sphincterotomy, which widens the opening of the sphincter of Oddi, allowing bile to flow more freely. In some instances, stenting of the bile duct may also be necessary to maintain patency and drainage [13-18]. Instances of endoscopic clipping of a duodenal diverticulum as a treatment for recurrent cholangitis due to biliary compression in Lemmel's syndrome have shown promising results [16]. In cases where endoscopic management proves insufficient, surgical intervention may become necessary. Sergi et al. document a noteworthy instance where retrograde cholangiopancreatography failed to achieve sphincterotomy, leading to the decision to perform a laparoscopic rendez-vous, highlighting the critical role of surgical strategies in managing complications when less invasive measures are not viable [19].

The prognosis for Lemmel syndrome is generally favorable with prompt diagnosis and treatment [9]. Endoscopic intervention offers a minimally invasive and effective approach to relieving the obstruction and alleviating symptoms. In this patient’s case, the sphincterotomy and stent placement successfully resolved the obstruction and her pain, leading to a successful recovery.

## Conclusions

The presented case emphasizes the importance of diagnostic vigilance and the use of advanced imaging techniques when investigating suspected biliary obstruction without an apparent cause. Although uncommon, Lemmel syndrome should be considered a potential diagnosis when biliary dilation is observed in the absence of calculi or mass lesions. MRCP and CT imaging played a crucial role in identifying the PAD compressing the distal bile duct, confirming the diagnosis of Lemmel syndrome. These imaging techniques not only detected the structural anomaly but also clarified its impact on bile flow, leading to the successful treatment of the patient’s symptoms through endoscopic sphincterotomy and biliary stenting. This case underscores the significance of maintaining a broad differential diagnosis and the appropriate use of advanced imaging modalities in evaluating biliary obstruction, as familiarity with the imaging findings associated with Lemmel syndrome is essential for timely and accurate diagnosis, enabling prompt intervention and favorable clinical outcomes.
